# Polyketide synthases of *Diaporthe helianthi* and involvement of *DhPKS1* in virulence on sunflower

**DOI:** 10.1186/s12864-017-4405-z

**Published:** 2018-01-06

**Authors:** Michelina Ruocco, Riccardo Baroncelli, Santa Olga Cacciola, Catello Pane, Maurilia Maria Monti, Giuseppe Firrao, Mariarosaria Vergara, Gaetano Magnano di San Lio, Giovanni Vannacci, Felice Scala

**Affiliations:** 1 Istituto per la Protezione Sostenibile delle Piante, CNR-IPSP, Via Università 133, 80055 Portici (Naples), Italy; 2 Université de Brest, EA 3882, Laboratoire Universitaire de Biodiversité et Ecologie Microbienne, IBSAM, ESIAB, Technopôle Brest-Iroise, 29280 Plouzané, France; 3 Dipartimento di Agricoltura, Alimentazione e Ambiente, Università di Catania, 95123 Catania, Italy; 4 Consiglio per la ricerca in agricoltura e l’analisi dell’economia agraria, Centro di ricerca Orticoltura e Florovivaismo, sede di Pontecagnano, via Cavalleggeri 25, 84098 Pontecagnano (Salerno), Italy; 5 Dipartimento di Scienze AgroAlimentari, Ambientali e Animali, Università di Udine, via Scienze, Udine, Italy; 6 Scuola Normale Superiore di Pisa, 56126 Pisa, Italy; 7 Dipartimento di Scienze Agrarie, Alimentari e Agro-Ambientali, Università di Pisa, 56124 Pisa, Italy; 8 Dipartimento di Gestione dei Sistemi Agrari e Forestali, Università Mediterranea di Reggio Calabria, 89061 Reggio Calabria, Italy; 9 Dipartimento di Agraria, Università di Napoli Federico II, 80055 Portici (Naples), Italy

**Keywords:** Polyketide synthases, *Diaporthe helianthi*, Plant pathogen, Pathogen virulence, toxins

## Abstract

**Background:**

The early phases of *Diaporthe helianthi* pathogenesis on sunflower are characterized by the production of phytotoxins that may play a role in host colonisation. In previous studies, phytotoxins of a polyketidic nature were isolated and purified from culture filtrates of virulent strains of *D. helianthi* isolated from sunflower. A highly aggressive isolate (7/96) from France contained a gene fragment of a putative nonaketide synthase (*lovB*) which was conserved in a virulent *D. helianthi* population.

**Results:**

In order to investigate the role of polyketide synthases in *D. helianthi* 7/96, a draft genome of this isolate was examined. We were able to find and phylogenetically analyse 40 genes putatively coding for polyketide synthases (PKSs). Analysis of their domains revealed that most PKS genes of *D. helianthi* are reducing PKSs, whereas only eight lacked reducing domains. Most of the identified PKSs have orthologs shown to be virulence factors or genetic determinants for toxin production in other pathogenic fungi. One of the genes (*DhPKS1*) corresponded to the previously cloned *D. helianthi lov*B gene fragment and clustered with a nonribosomal peptide synthetase (NRPS) -PKS hybrid/lovastatin nonaketide like *A. nidulans* LovB. We used *DhPKS1* as a case study and carried out its disruption through *Agrobacterium*-mediated transformation in the isolate 7/96. *D. helianthi DhPKS1* deleted mutants were less virulent to sunflower compared to the wild type, indicating a role for this gene in the pathogenesis of the fungus.

**Conclusion:**

The PKS sequences analysed and reported here constitute a new genomic resource that will be useful for further research on the biology, ecology and evolution of *D. helianthi* and generally of fungal plant pathogens.

**Electronic supplementary material:**

The online version of this article (doi:10.1186/s12864-017-4405-z) contains supplementary material, which is available to authorized users.

## Background

Polyketides are a large and diverse group of secondary metabolites with different biological activities, including pathogenicity, such as T-toxin produced by *Cochliobolus heterostrophus* [1] and melanin, a pigment essential for plant pathogenesis by many fungi [2, 3]. Biosynthesis of these metabolites is accomplished by polyketide synthases [4]. Polyketide synthases (PKSs) are modular enzymes classified on the basis of their molecular architecture and operating mechanisms into types I, II and III [5–8]. Fungal PKs show a structural diversity that can vary from simple aromatics to highly modified complex reduced-type compounds [9]. Many fungal PKSs have an architecture based on single modular iterative type I polyketide synthases (iPKSs), containing ketosynthase (KS), acyltransferase (AT) and acyl carrier protein (ACP) domains. In addition to these, other functional domains, such as ketoreductase (KR), dehydratase (DH), enoylreductase (ER), methyltransferase (MeT) and thioesterase (TE), may be present in PKSs [10].

*Diaporthe helianthi* Munt.-Cvetk., Mihaljč. & M. Petrov (syn. *Phomopsis helianthi* Munt.-Cvetk., Mihaljč. & M. Petrov) is a phytopathogenic fungus which causes stem canker and leaf shedding in sunflower (*Helianthus annuus* L.). The fungus invades and spreads through the leaves, progresses towards the petioles along foliar veins, and finally enters the stem, where cankers form in the advanced stage of pathogenesis [11]. *D. helianthi* is an important pathogen with a worldwide distribution. First reported in the former Yugoslavia [12], it subsequently spread to several other countries [13–15]. It can cause significant losses in yield and reduction of oil content when environmental conditions are favourable for disease development [16]. In Italy this disease has been observed since 1987, but even when climatic conditions are favourable to the disease [17, 18], its appearance is sporadic [19]. Epidemiological differences of this widespread disease could be explained by a high genetic diversity occurring in the *D. helianthi* populations depending on their geographic origin. Intraspecific variability has been previously displayed by isolates representative of diverse sunflower-growing areas [20–23]. All the isolates collected in France and in the former Yugoslavia, where epidemics of sunflower stem canker are severe, formed a monophyletic clade clearly distinct from all other isolates, while all the Italian isolates were phylogenetically distant from this cluster, evidencing a clear link between genetic biotype and pathogenic behaviour [21].

It has been nowadays accepted that sunflower stem canker is associated with a complex of *Diaporthe* species with different levels of pathogenicity. The main causal agent of the disease has been identified as *D. gulyae* sp. nov. in association with two less virulent species, *D. kochmanii* sp. nov. and *D. kongii* sp. nov. in Australia [23], whereas in the United States, *D. gulyae* and *D. helianthi* were both identified as causal agents of the disease having similar levels of aggressiveness [24].

The highly virulent French isolate 7/96 can be referred as *D. helianthi* sensu stricto. A DNA region of 532 bp from this isolate, shared with all highly virulent strains but not with the Italian mildly virulent isolates, was identified and sequenced [25]. This sequence, named lovB (accession number: AJ512137) showed a high similarity to genes encoding polyketide synthases (PKSs) from several species of filamentous fungi, including *mlcA* and *mlcB* of *Penicillium citrinum*, *lovB* of *Aspergillus terreus*, *fum5* of *Gibberella moniliformis*, *pks1* of *Cochliobolus heterostrophus* and *pks1* of *G. fujikuroi*.

The mechanisms of pathogenicity and symptom induction are poorly understood in *D. helianthi*. Mazars et al. [26, 27] have demonstrated the production of a polyketidic phytotoxin, named phomozin, during pathogenesis on sunflower leaves and in culture filtrates of a French *D. helianthi* strain. The purified toxin produced symptoms comparable to those caused by pathogen infection. Subsequently, Avantaggiato et al. [28] purified other two phytotoxic metabolites, identified as *cis*- and *trans*-4,6-dihydroxymellein, from cultures of French and Italian isolates with different degrees of virulence. These toxins show a structure similar to phomozin, sharing with it the same precursor, known as orsellinic acid [28].

In the present study we generated a draft genome of the highly virulent isolate *D. helianthi* 7/96 and annotated 40 genes coding for putative PKSs [29]. By using the Pathogen-Host Interaction database (PHI-base) (http://www.phi-base.org) [30], orthologs of genes known to be involved in biosynthesizing PKs, which are virulence factors in other fungal species, were identified. Furthermore, the role in virulence of the gene *DhPHS1* (= *D. helianthi* polyketide synthase 1) was evaluated through a gene disruption approach.

## Methods

### Fungal isolate and media

*D. helianthi* highly virulent French isolate 7/96 belongs to the fungal collection of Department of Agriculture, Food and Environment, University of di Pisa (Italy) [25] and was maintained on slants of PDA (potato dextrose agar, Difco) under mineral oil at 4 °C.

### Diaporthe *helianthi* strain 7/96 draft genome sequence v2

Based on raw data available from a previous project [29], we generated a new *D. helianthi* strain 7/96 genome assembly. Paired end reads of 90 bp (1.80 Gbp) were assembled using SPAdes 3.11.0 [31]. The genome of *D. helianthi* consists of 7376 sequence scaffolds with a total assembly length of 63.67 Mbp (N50 = 20,184 and L50 = 860), 43.99% GC-content, and a maximum scaffold size of 151,286 bp. The completeness of the assembly was assessed using BUSCO v12 [32], which estimated the genome sequence to be 99.65% complete. The genome was annotated using the MAKER2 pipeline [33]. Overall, 13,139 protein-coding gene models were predicted.

The new genome assembly of *D. helianthi* strain 7/96 is present in GenBank with accession number: MAVT00000000.2.

### Genomic characterization of putative PKSs genes

Putative PKS genes were identified according to Klarsson et al. [34] and manually inspected for conserved domain (acyl transferase [AT] – InterPro domain IPR014043, acyl carrier protein or phosphopantetheine attachment site [ACP or PP] – IPR009081, beta-ketoacyl synthase N-terminal domain [KS-N] – IPR014030, beta-ketoacyl synthase C-terminal domain [KS-C] – IPR014031, Ketoreductase [KR] – IPR013968, polyketide synthase dehydratase [DH] – IPR020807, polyketide synthase, enoylreductase domain [ER] – IPR020843, methyltransferase [MT] – IPR013217, thioesterase [TE] – IPR001031) using InterProScan [35]. Moreover, putative *D. helianthi* PKSs sequences were aligned with references of other ascomycetes using MAFFT 7.310 [36] and a phylogenetic analysis was performed with PhyML 3.0 [34].

### DNA molecular techniques

Total DNA was obtained from *D. helianthi* grown on PDA plates overlaid with a cellophane membrane. Plates were inoculated with mycelial plugs and incubated at 24 °C for 5 days. Mycelium mats were peeled from membranes, freeze-dried overnight, and used for DNA extraction according to Raeder and Broda [37].

Plasmids were purified from *E. coli* DH5α cultures, grown on LB (yeast extract 5 g/L, NaCl 5 g/L, tryptone 10 g/L) by using QIAprep Spin Miniprep Kit (QIAGEN).

All PCR amplifications were performed as follows: initial denaturation at 94 °C for 3 min, 35 cycles of denaturation (45 s at 94 °C), annealing (45 s at 60 °C) and extension (1 min 50 s at 72 °C), and a final extension at 72 °C for 10 min. PCR reactions were carried out in PCR buffer (Promega, Madison, WI, USA), 0.2 mM dNTPs (Roche Applied Science, Mannheim, Germany), 0.2 M each primer, 0.4 unit of Taq polymerase (Promega), and ca. 5 ng of template DNA.

To amplify the complete sequence of *DhPKS1* gene, PCR were performed by using Platinum® Taq DNA Polymerase High Fidelity (ThermoFisher) with the primers *DhPKS1for* (ATGTCCAAGGCAATTTGTACTAC) and *DhPKS1rev* (CCTATCGCTAACAATCTTGT). The PCR cycles were as follows: initial denaturation at 95 °C for 5 min, 35 cycles of denaturation (45 s at 95 °C), annealing (45 s at 60 °C) and extension (8 min at 68 °C), and a final extension at 68 °C for 10 min.

Sequence editing and analysis were carried out using BioEdit 7.0.5.2 (http://www.mbio.ncsu.edu/bioedit/bioedit.html) software and online tools available at European Bioinformatics Institute (http://www.ebi.ac.uk/).

DNA restriction, elution and ligation, and Southern blot analysis were carried out as described by [38], performing hybridization at 60 °C for 16 h and washing of blots in 2× SSC and 0.5 SSC at room temperature.

### Construction of plasmid pUR5750*∙ΔDhPKS1*

**Fig. 1:**
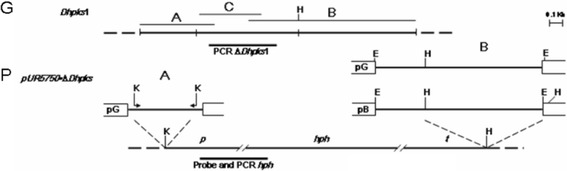
(G): Restriction map (not all restriction sites are included) of the genomic fragment of *Diaporthe helianthi* containing the *DhPKS*1 gene with the original sequence isolated by Vergara et al. (2004) (C) plus its flanking gene portions (A and B) used for hygromicin phosphotransferase (HPH) gene replacement cassette construction. (P): Restriction map (not all sites are included) of plasmid pUR5750•Δ*DhPKS*1 containing *hph* gene of *E. coli* under the control of the *A. nidulans gpd*A promoter (*p*) and *trpc* terminator (*t*). In the construction design, pGEM-T Easy vector system (pG) and pBluescript KS (pB) were used as intermediate vectors. H = *Hind*III; K = *Kpn*I; E = *Eco*RI

For *D. helianthi* transformation, the binary vector pUR5750, described by De Groot et al. [39] was used. This vector contains the neomycin phosphotransferase gene under the control of the nopaline synthase promoter and *trpC* terminator that confers resistance to kanamycin; it also carries, between the *Hind*III and *Kpn*I restriction sites, the *E. coli* hygromycin B (*hph*)-resistance cassette, coding for the hygromycin B phosphotransferase enzyme (*hph*) under the control of the *A. nidulans gpdA* promoter and *trpC* terminator. The two flanking regions of the previously isolated sequence *LovB* [25] were identified and cloned with GenomeWalker kit (Clontech Laboratories, Palo Alto, CA) as follows: Separate fungal DNA aliquots were digested with four different restriction enzymes (*EcoR*V, *Dra*I, *Pvu*II, *Stu*I) leaving blunt ends and ligated to adaptors. For each fragment library, two primary PCR amplifications were carried out using an adaptor primer provided with the kit and an outer, gene-specific primer for downstream and upstream walking, 5′-AAG GTG GAC ACG GCA TAC CAC TCA TT-3′ and 5′-CCA AGT CTT CAG CAG GAA TAT CAA CCA C-3′, respectively. The primary PCR product was then diluted and used as a template for a secondary PCR amplification using a nested adaptor primer and nested gene-specific primers (5′-AGC TGC AAG TGC CTT ACC ACG GAT TAC-3′ for downstream walking and 5′-ATG AGT GGT ATG CCG TGT CCA CCT TC-3′ for upstream walking). The resulting DNA, flanking *LovB*, of 548 and 1256 bp were singly cloned in p-GEM-T Easy vector system (Promega) to form clones and DL2, respectively. To generate pUR5750∙*ΔDhPKS1*, the two previously cloned regions were excised from p-GEM-T Easy vector and fragments of about 548 bp (5′ flanking gene fragment, called hereafter “A”) and 944 bp (3′ flanking gene fragment, called hereafter “B”), were inserted in pUR5750 *Kpn*I and *Hind*III restriction sites, respectively, at the sides of (*hph*)-resistance cassette (Fig. 1).

In detail, the A fragment was excised from the plasmid by EcoRI digestion, and PCR-amplified with specific primers (5′AGGTACCATTCGATTACTATAGGGCACG 3′ and 5′AGGTACC GTACTCAGGCATGGAGCAAA 3′), carrying at ends the KpnI cutting site sequence. The B fragment, was excised from the plasmid pGEM-T easy by EcoRI digestion, and inserted in the *Eco*RI site of pBluescript KS; from this construct a smaller fragment of about 944 bp was excised, with HindIII restricion enzyme. The two *DhPKS1* gene fragments (KpnI 548 bp and HindIII 944 bp) flanking the designed knock-out site, were inserted in the corresponding cutting-site in the plasmid pUR5750, upstream and downstream of the *hph* resistance cassette, to form a new plasmid named pUR5750*∙ΔDhpks1*. Finally, pUR5750∙Δ*DhPKS1* was transferred into *A. tumefaciens* LBA1100 by electroporation [40]; electroporation conditions were 25 μF, 200 Ω, 2.5 kV (0.2 cm cuvettes) in Gene Pulser® electroporator (Bio-Rad, USA), and transformants were selected on LB agar with and kanamycin (100 μg/ml). *Agrobacterium* strains containing the binary vectors were identified by PCR.

### *Agrobacterium tumefaciens*-mediated gene disruption

The LBA1100-derived strain transformed with pUR5750∙Δ*DhPKS1* was grown at 28 °C for 3 days in Petri dishes containing LB medium supplemented with kanamycin (100 μg/ml). One hundred milliliter of liquid LB supplemented with kanamycin (100 μg ml^−1^) were inoculated with a single bacterial colony and incubated at 28 °C overnight on an orbital shaker at 150 rpm. Bacterial cells were harvested by centrifugation at 12000 *g* at 4 °C and re-suspended in 5 ml of IM (1 mM KH_2_PO_4_ pH 4.8; 2.4 mM MgSO_4_•7H_2_O; 5 mM NaCl; 0.068 mM CaCl_2_; 0.003 mM FeSO_4_•7H_2_O; 0.0015 mM ZnSO_4_•7H_2_O; 0.002 mM CuSO_4_ 5H_2_O; 0.008 mM H_3_BO_3_; 0.003 mM MnSO_4_•H_2_O; 0.002 mM Na_2_MoO_4_•2H_2_O; 6.25 mM NH_4_NO_3_; 54.2 mM glycerol; 40 mM 2-(N-morpholino)ethanesulfonic acid; 0.05 mM glucose). The bacterial suspension was distributed in aliquots of 1 ml and further incubated at 30 °C for 6 h under stirring. Cultures of the fungal isolate 7/96b were grown on IM-agar supplemented with acetosyringone (AS, 0.2 mM) and incubated at 24 °C. After 4–5 days, mycelial plugs of these colonies were used to inoculate 10 ml of liquid IM + AS. The cultures were then incubated for 4 days at 24 °C under gentle stirring and periodically vortexed for 5 min. The mycelium was recovered by centrifugation at 4000 *g* for 15 min and re-suspended in 5 ml of IM + AS.

For co-cultivation, 1 ml of mycelial suspension was mixed with an equal volume of bacterial suspension at 24 °C and stirred for about 20 min. The mix was plated onto nitrocellulose filters on a co-cultivation medium (IM-agar + AS) and incubated at 24 °C for 3 days. After growth on co-cultivation medium, the filters were transferred to PDA amended with hygromycin (25 μg/ml) as selective medium for fungal transformants and cefotaxime (200 μM) to inhibit growth of *A. tumefaciens*, and incubated at 24 °C for 20 days. Hygromicin resistant colonies were purified by three successive hyphal transfers on PDA supplemented with 100 μg/ml hygromicin to select hyphae containing only transformed nuclei. The analysis of transformants was performed by PCR using specific primers designed to amplify both hygromicin resistance cassette (*hph2for*: 5′-ATGGCAACAAATGTTGGACTG-3′; *gpdArev*: 5′-CAAGGAGGAGTAAGCTCCTT-3′), and *DhPKS1* gene replacement site (*Dhpks537for*: 5′-GTACTCCAAGGCTTTATCGC-3′; *Dhpks1326rev:* 5′-TGATGTAGAACTGGGCCACA-3′) of *D. helianthi*. As control, *D. helianthi* mutants containing pUR5750 empty vector insertion were obtained.

Single copy insertion was verified by Southern blot analysis of genomic DNA digested with *Spe*I and *Bgl*II restriction enzymes, which do not cut in the Agrobacterium-transferred construct. Hybridization was carried out with digoxigenin-labelled probe obtained by PCR-amplification of binary vector *pUR5750ΔDhPKS1* plasmidic DNA using *hph2for - gpdArev* primers. *D. helianthi* wild type DNA and PCR-amplified construct *ΔDhPKS1* containing the hygromycin B (hph)-resistance cassette, were used as negative and positive controls, respectively.

### Phytotoxicity and virulence assays

For phytotoxicity tests, mycelial plugs from actively growing colonies of *D. helianthi* isolate 7/96 and transformants were grown, in static condition, on liquid medium in 2 L volume Erlenmeyer flasks containing 1 L of substrate made with: 3 g/L L-asparagine, 15 g/L sucrose, 1 g/L K_2_HPO_4_, 0.5 g/L MgSO_4_7 H_2_O, 0.5 g/L KCl, 0.018 g/L FeSO_4_7H_2_O, 5 g/L fresh sunflower tissue. The same liquid medium without fungal inoculation was used as a control. After 28 days, the liquid cultures were vacuum filtered, sterilised through Millipore filters of 0.2 μm and used in phytotoxicity tests, performed with cuttings of 15-day-old sunflower seedlings according to Avantaggiato et al. [28]. Ten sunflower cuttings were used per experiment and the experiment was replicated three times.

For virulence tests inoculum was prepared by growing *D. helianthi* strain 7/96 and transformants in Petri dishes containing different parts of sunflower (stems, leaves and flowers). Fresh plant tissues, surface sterilized with sodium hypochlorite (2.5%/vol.) and washed in sterile water, were dried, chopped (250 g/L) and mixed with agar (15 g/L). The fungus inoculated on the sunflower-agar substrate was left to grow in the dark for 1 week at 25 °C. Seventy-eight-day-old sunflower plants (very susceptible cv Ala) at the flower bud stage were inoculated by placing a mycelial plug both on the upper surface of leaves and at the insertion point of the leaf petiole (sticking with a wooden toothpick). The diseased area was measured recording two diameters of the necrotic spot, by using a digital caliper. Inoculated plants were kept in a moist chamber for 7 days at 24 °C. The bioassay was carried out on five plants for each fungal strain and was repeated twice. Data were analysed by ANOVA test with Welsch modification, because data were normally distributed but not homoscedastic. Different means were separated by T3 Dunnett test. Statistical analyses were performed by SPSS 20.0 software.

## Results

### PKS genes in *Diaporthe helianthi* genome

**Fig. 2:**
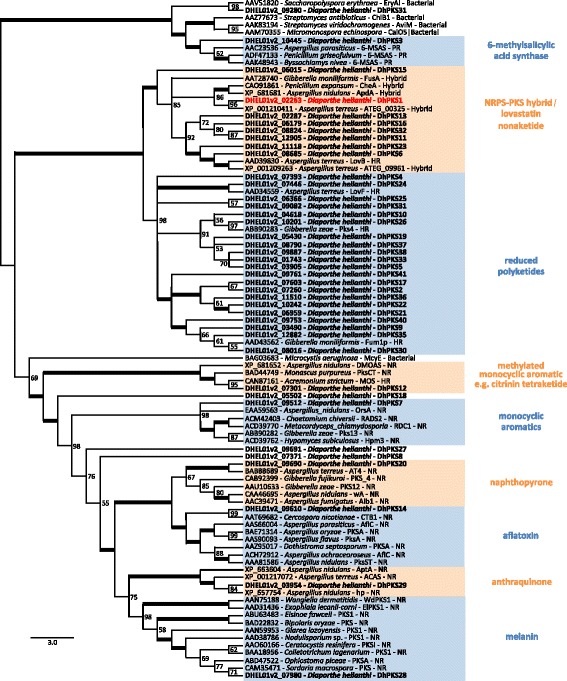
Phylogenetic tree of *Diaporthe helianthi* PKSs proteins (highlighted in bold) and reference PKSs from other fungi based on Karlsson et al., [34] . DhPKS1 used for molecular characterization is highlighted in red. Numbers next to the nodes represent bootstrap values. Full *D. helianthi* PKS genes information is provided in Additional file 1

By searching the draft nuclear genome of *D. helianthi* isolate 7/96 [29], we found a large number of putative PKS homologues which have been deposited in GenBank (Additional file 1). Most *PKS* genes coded by *D. helianthi* were reducing PKSs, whereas only eight PKSs lacked reducing domains and clustered with non-reducing PKSs (Additional file 1). DhPKS8, DhPKS18, DhPKS27 proteins did not cluster into any of the clades indicated in Fig. 2. DhPKS1 was placed in nonribosomal peptide synthetase (NRPS)-PKS clade, with the closest sequence being ATEG_00325 protein from *A. terreus* involved in isoflavipucine biosynthesis.

### Homologies of *DhPKSs* with other genes involved in host-pathogen interaction

By whole genome sequencing of *D. helianthi*, we were able to decipher the complete sequence of all *DhPKS* genes and in silico deduced amino acid sequences (Additional file 1). Through a PHI-base interrogation we identified homologs for all *DhPKS* genes, except for two (*DhPKS11* and *DhPKS35*), with experimentally verified pathogenicity, virulence and effector genes from fungal, oomycete and bacterial pathogens, infecting animal, plant, fungal and insect hosts. Results of this search are reported in Table 1.

**Table 1:** List of all *DhPKS* genes, with the exclusion of two (*DhPKS11* and *DhPKS35*), with experimentally verified pathogenicity, virulence and effector genes from fungal, oomycete and bacterial pathogens, which infect animal, plant, fungal and insect hosts, obtained by searching PHI-base (http://www.phi-base.org)

PHI Closest hits (proteins)									
Name	GenBank Acc. Num.	Putative protein length (aa)	Organism	Disease	Protein name	Protein ID	Identity (%)	Similarity (%)	Ref.
DhPKS1	KR153185	2501	*M. oryzae*	Rice blast	ACE1	Q6ZX14	33.98	50.56	[50]
			*B. cinerea*	Gray mould	BcBOA6	B1GVX7	34.76	53.57	[44]
			*A. brassicicola*	Black spot	DEP5	D2E9X0	32.68	50.45	[52]
DhPKS2	KR153184	2635	*G. fujikuroi*	Foolish seedling	FUM1 (FUM5)	Q9Y8A2	32.53	49.48	[66]
			*C. heterostrophus*	Leaf blight	PKS1	Q92217	31.58	48.37	[3]
DHPKS3	KR153183	1798	*A. oligospora*	Nematophagous fungus	AoMls	G1XLT2	46.59	63.93	[67]
DHPKS4	KR153182	3071	*F. verticillioides*	Ear and stalk rot of maize	FVEG_12528	W7NCN7	28.69	45.46	[68]
			*F. graminearum*	Fusarium ear blight	PKS4 (ZEA1)	Q2VLJ2	28.14	45.46	[69]
DHPKS5	KR153181	2585	*C. heterostrophus*	Leaf blight	PKS1	Q92217	33.22	51.15	[3]
			*G. fujikuroi*	Foolish seedling	FUM1 (FUM5)	Q9Y8A2	31.92	48.97	[66]
DHPKS6	KR153180	3013	*M. oryzae*	Rice blast	ACE1	Q6ZX14	35.17	53.24	[50]
			*B. cinerea*	Gray mould	BcBOA6	B1GVX7	38.13	56.32	[44]
DHPKS7	KR153179	2140	*C. graminicola*	maize anthracnose	CgPKS1	C9W7X1	32.98	49.38	[70]
			*F. graminearum*	Fusarium ear blight	PKS13 (ZEA2)	Q2VLJ3	36.24	55.66	[71]
			*A. alternata*	Leaf spot	ACRTS2	F8R4Y0	36.75	53.86	[63]
DHPKS8	KR153178	2193	*A. alternata*	Leaf spot	ACRTS2	F8R4Y0	38.87	55.41	[63]
			*C. neoformans*	Facultative plant and animal fungal pathogen	Cln1	J9VV14	38.57	55.52	[72]
			*A. fumigatus*	Respiratory disease	ALB1	O59897	38.17	56.14	[73]
			*A. fumigatus*	Respiratory disease	PKSP	O60026	38.08	56.09	[74]
			*C. lagenarium*	Anthracnose	PKS1	P79068	37.07	54.01	[62]
			*E. dermatitidis*	Phaeohyphomycosis	WdPKS1	Q9Y7A7	36.64	53.72	[75]
DHPKS9	KR153177	2519	*G. fujikuroi*	Foolish seedling	FUM1 (FUM5)	Q9Y8A2	34.21	51.88	[66]
DHPKS10	KR153176	2209	*F. verticillioides*	Ear and stalk rot of maize	FVEG_12528	W7NCN7	38.91	54.24	[68]
			*C. heterostrophus*	Leaf blight	PKS1	Q92217	38.05	54.73	[3]
DhPKS11	KR153175	171							
DHPKS12	KR153174	2492	*A. alternata*	Leaf spot	ACRTS2	F8R4Y0	31.21	47.02	[63]
DHPKS13	KR153173	4088	*M. oryzae*	Rice blast	ACE1	Q6ZX14	39.13	57.03	[50]
			*B. cinerea*	Gray mould	BcBOA6	B1GVX7	39.88	57.66	[44]
DHPKS14	KR153172	2191	*A. fumigatus*	Respiratory disease	ALB1	O59897	42.51	60.58	[73]
			*A. fumigatus*	Respiratory disease	PKSP	O60026	42.46	60.48	[74]
			*C. nicotianae*	Leaf spot	CTB1	Q6DQW3	42.97	59.11	[64]
			*C. sativus*	Spot blotch disease	PKS1	G8DNT0	39.75	58.54	[76]
			*A. alternata*	Leaf spot	ACRTS2	F8R4Y0	39.62	57.97	[63]
			*C. neoformans*	Facultative plant and animal fungal pathogen	Cln1	J9VV14	38.06	56.65	[72]
DHPKS15 (partial)	KR153171	1613	*B. cinerea*	Gray mould	BcBOA6	B1GVX7	34.61	50.99	[44]
DHPKS16	KR153170	3886	*M. oryzae*	Rice blast	ACE1	Q6ZX14	34.17	52.24	[50]
			*B. cinerea*	Gray mould	BcBOA6	B1GVX7	33.69	52.09	[44]
DHPKS17	KR153169	2616	*G. fujikuroi*	Foolish seedling	FUM1 (FUM5)	Q9Y8A2	31.55	48.7	[66]
			*A. brassicicola*	Black spot	DEP5	D2E9X0	36.67	53.72	[52]
DHPKS18	KR153168	2161	*F. graminearum*	Fusarium ear blight	PKS13 (ZEA2)	Q2VLJ3	29.11	45.31	[71]
DHPKS19	KR153167	2647	*G. fujikuroi*	Foolish seedling	FUM1 (FUM5)	Q9Y8A2	32.46	50.09	[66]
DHPKS20	KR153166	1996	*A. fumigatus*	Respiratory disease	ALB1	O59897	44.37	60.66	[73]
DHPKS21 (partial)	KR153165	814	*A. brassicicola*	Black spot	DEP5	D2E9X0	41.09	57.14	[52]
DHPKS22	KR153164	2505	*A. brassicicola*	Black spot	DEP5	D2E9X0	32.25	50.16	[52]
DHPKS23 (partial)	KR153163	7787	*B. cinerea*	Gray mould	BcBOA6	B1GVX7	53.44	68.48	[37]
DHPKS24	KR153162	2545	*C. heterostrophus*	Leaf blight	PKS1	Q92217	32.44	50.48	[3]
DHPKS25	KR153161	2590	*F. verticillioides*	Ear and stalk rot of maize	FVEG_12528	W7NCN7	35.75	53.85	[68]
			*F. graminearum*	Fusarium ear blight	PKS4 (ZEA1)	Q2VLJ2	34.1	50.4	[71]
			*C. heterostrophus*	Leaf blight	PKS1	Q92217	32.44	50.48	[3]
DHPKS26	KR153160	2543	*F. graminearum*	Fusarium ear blight	PKS4 (ZEA1)	Q2VLJ2	36.73	53.29	[71]
			*F. verticillioides*	Ear and stalk rot of maize	FVEG_12528	W7NCN7	34.6	51.13	[68]
			*C. heterostrophus*	Leaf blight	PKS1	Q92217	36.7	52.34	[3]
DHPKS27	KR153159	2064	*A. alternata*	Leaf spot	ACRTS2	F8R4Y0	47.12	64.5	[63]
DHPKS28	KR153158	2169	*C. lagenarium*	Anthracnose	PKS1	P79068	71.73	83.2	[62]
			*C. graminicola*	maize anthracnose	CgPKS1	C9W7X1	32.98	49.38	[70]
			*C. sativus*	Spot blotch disease	PKS1	G8DNT0	47.89	65.35	[76]
			*A. alternata*	Leaf spot	ACRTS2	F8R4Y0	47.12	64.5	[63]
DHPKS29	KR153157	1813	*P. nodorum*	Septoria nodorum blotch	SnPKS19	Q0TX07	45.31	61.45	[77]
			*C. graminicola*	maize anthracnose	CgPKS1	C9W7X1	32.98	49.38	[70]
DHPKS30	KR153156	2624	*G. fujikuroi*	Foolish seedling	FUM1 (FUM5)	Q9Y8A2	41.46	57.69	[66]
			*A. brassicicola*	Black spot	DEP5	D2E9X0	36.4	53.12	[52]
DHPKS31	KR153155	2753	*A. oligospora*	Nematophagous fungus	AoMls	G1XLT2	46.59	63.93	[67]
DHPKS32	KR153154	2484	*M. oryzae*	Rice blast	ACE1	Q6ZX14	39.74	56.57	[50]
			*B. cinerea*	Gray mould	BcBOA6	B1GVX7	37.15	55.25	[44]
DHPKS33	KR153153	2254	*G. fujikuroi*	Foolish seedling	FUM1 (FUM5)	Q9Y8A2	28.72	45.55	[66]
DHPKS34	KR153152	2365	*C. heterostrophus*	Southern corn leaf blight	PKS2	Q6RKG2	32.00	49.87	[1]
			*C. heterostrophus*	Leaf blight	PKS1	Q92217	36.83	54.98	[3]
			*F. verticillioides*	Ear and stalk rot of maize	FVEG_12528	W7NCN7	36.16	53.24	[68]
			*F. graminearum*	Fusarium ear blight	PKS4 (ZEA1)	Q2VLJ2	35.14	52.15	[71]
DhPKS35	KR153151	441							
DHPKS36	KR153150	2422	*A. brassicicola*	Black spot	DEP5	D2E9X0	51.48	66.93	[52]
DHPKS37	KR153149	2597	*C. heterostrophus*	Leaf blight	PKS1	Q92217	34.46	53.1	[3]
			*G. fujikuroi*	Foolish seedling	FUM1 (FUM5)	Q9Y8A2	33.81	51.81	[66]
DHPKS38	KR153148	2652	*C. heterostrophus*	Leaf blight	PKS1	Q92217	32.04	49.81	[3]
DHPKS39	KR153147	2511	*G. fujikuroi*	Foolish seedling	FUM1 (FUM5)	Q9Y8A2	38.11	56.07	[66]
DHPKS40	KR153146	2380	*A. brassicicola*	Black spot	DEP5	D2E9X0	30.69	47.58	[52]
			*F. verticillioides*	Ear and stalk rot of maize	FVEG_12528	W7NCN7	33.2	50.3	[68]

### Replacement of *DhPKS1*

Using oligonucleotides designed at 5′ and 3′ ends of genomic sequence of *DhPKS1* we sequenced the complete gene (7877 bp) confirming its in silico prediction. In silico analysis showed that the coded protein corresponded to a highly reducing (HR) type I iPKS, containing the full set of domains, ketoacyl synthase (KS), acyl transferase, (AT), ketoreductase (KR), dehydratase (DH) and acyl carrier protein (ACP) (Additional file 1). Due to the identity of *DhPKS1* with *lovB* (AN AJ512137) fragment identified by Vergara et al. [25] we decided to test its potential involvement in pathogenicity of *D. helianthi*.

**Fig. 3:**
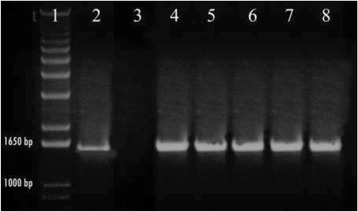
PCR analysis of *Diaporthe helianthi* strain 7/96 (lane 3) and *ΔDhPKS1* putative transformants (lanes 4–8) with *hph*2-for/*trpc*2-rev primers. Lanes 1 and 2 correspond to 1 kb molecular weight ladder and positive control, respectively

**Fig. 4:**
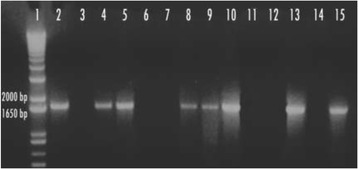
PCR analysis of *Diaporthe helianthi* strain 7/96 (lane 2) and *ΔDhPKS1* putative transformants (lanes 4–15) with *Dhpks537*-for/*Dhpks1326*-rev primers. Lanes 1 and 3 correspond to 1 kb molecular weight ladder and negative control, respectively

**Fig. 5:**
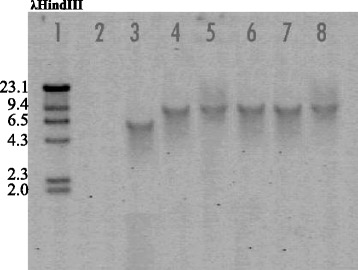
Southern blot analysis of *ΔDhPKS1* transformants (lanes 4–8) and *Diaporthe helianthi* strain 7/96 (lane 2) genomic DNA digested with *Spe*I + *Bgl*II, by hybridization with a hygromycin-resistance cassette specific probe. Lanes 1, 2 and 3 correspond to λHindIII molecular weight ladder, negative and positive controls, respectively

Transformation of *D. helianthi* 7/96 with the gene disruption cassette in *DhPKS1* yielded 160 hygromicin-resistant colonies. These putative transformants were purified by successive transfers of mono-hyphal and single protoplasts to selective medium amended with hygromycin 100 μg/L. Hygromycin resistance of transformants was not lost upon successive transfers; 74 of the hygromicin resistant colonies were screened by two PCR analyses. The first PCR was performed on the genomic DNA of each transformant, with the *hph2for – trpc2rev* primers specific to amplify the hygromicin resistance cassette (Fig. 3). *Hph*-containing transformants were then screened with a second PCR, to verify the homologous integration of the construct. In this case, we used *Dhpks537for - Dhpks1326rev* primers, designed on sequences internal to the replacement site of *DhPKS1* (Fig. 4). This second PCR showed that 13 out of the 74 (18%) transformants integrated the *ΔDhPKS1* construct at the homologous site. The number of integrated copies of the construct in the genome of *D. helianthi* transformants was ascertained by Southern blot analysis with a probe specific for the hygromicin-resistance cassette (Fig. 5). Single copy integration of the construct occurred in five transformants designated as Tr1-5, as the enzymes used for genomic DNA digestion do not cut the *Agrobacterium*-transferred construct.

### Phytotoxicity and virulence bioassays

**Fig. 6:**
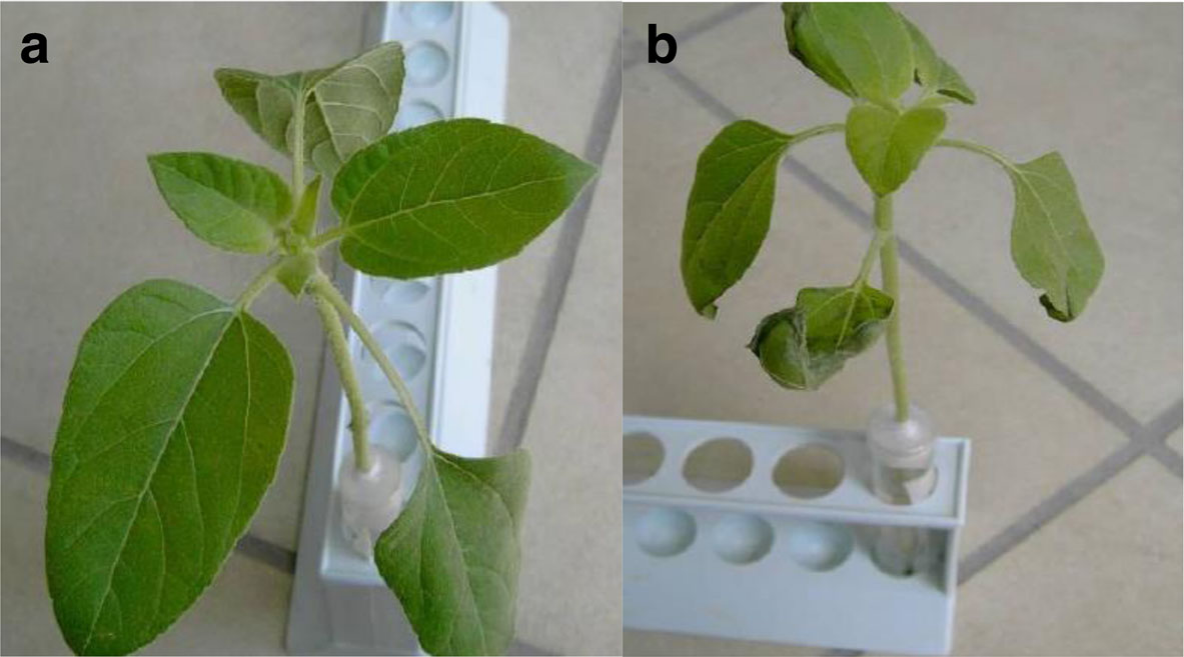
Phytotoxic effect of *ΔDhPKS1* mutant 7/96-Tr1 (**a**) and *Diaporthe helianthi* strain 7/96 (**b**) culture filtrates on sunflower cuttings

*ΔDhPKS1* mutants showed regular in vitro development and their growth rates were not significantly different from that of the wild type. Sunflower cuttings steeped in liquid cultures of the *D. helianthi* isolate 7/96 showed, after 5 days, brown marginal necrotic lesions and leaf chlorosis attributable to filtrate phytotoxicity. Cuttings steeped in culture extracts of *DhPKS1* knock-out mutants, did not show clear evidence of phytotoxicity (Fig. 6).

**Fig. 7:**
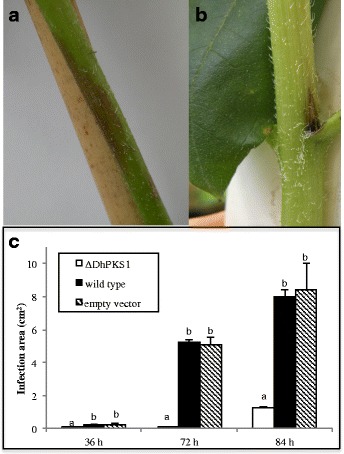
Pathogenicity bioassay of *Diaporthe helianthi* strain 7/96 (**a**) and *ΔDhPKS1* mutant 7/96-Tr1 (**b**) on sunflower stem. **c** Development of necrotic area (cm^2^) on leaf blade in sunflower plants inoculated with the mutant (ΔDhPKS1), parental (wild type) and empty vector transformant strains. Observations made at 36, 72 and 84 h after inoculum. Different letters indicate statistically significant differences (*p* < 0.01) at T3 Dunnett test

Inoculation of sunflower plants with *D. helianthi* strain 7/96 wild type resulted in typical symptoms of stem canker disease when observed 20 days after inoculation (Fig. 7a). Plants inoculated with transformants showed less intense symptoms (Fig. 7b). The disease severity in terms of necrotic area was measured in cm^2^ on leaf blade and stem, in sunflower plants inoculated with the parental strain and transformants. For each time point, infection was markedly less severe with *ΔDhPKS1* mutant than with the other strains. In particular the average necrotic area (cm^2^) at 84 h was 1.3 ± 0.28, 8.0 ± 1.65 and 8.5 ± 1.56, for *ΔDhPKS1*, wild type and empty vector transformant strains, respectively (Fig. 7c).

## Discussion

The management of sunflower stem canker requires the identification of diverse species of *Diaporthe* associated with it, the determination of their aggressiveness and the study of pathogenicity mechanisms. In this paper we analysed the genome of the highly aggressive isolate 7/96 of *D. helianthi*, regarded as the main causal agent of sunflower stem canker epidemics in Europe [25], on the basis of its putative polyketide synthase genes. We found in its genome at least 40 PKS genes, more than those reported in other ascomycetes: 15 in *G. moniliformis*, 16 in *G. zeae*, 20 in *B. cinerea*, 25 in *C. heterostrophus* [41] and 27 in *Aspergillus nidulans* [42]. Such a repertoire of PKSs makes possible the synthesis of almost all known types of polyketide compounds by *D. helianthi* 7/96.

The PHI-base search demonstrated that all the putative 40 PKS proteins found in *D. helianthi*, with the exception of DhPKS11 and DhPKS35, have at least one ortholog in other pathogenic fungi or bacteria (Table 1). The majority of the reported orthologous genes have been demonstrated to be important factors in pathogenicity or virulence by gene deletion experiments.

A fragment of *LovB*-like coding sequence was isolated by Vergara et al. [25] from the genome of the aggressive isolate of *D. helianthi* 7/96, and proved to be present in the genome of the most aggressive isolates of *D. helianthi*, such as the French and Yugoslavian ones. In the present work this gene, named *DhPKS1*, was fully characterized. In silico sequence analysis revealed that *DhPKS1* belongs to subclade II of type I reducing PKS [41], having the typical conserved domain of this protein family KS-AT-DH-(ME)KR-PP-(CON)-(AMP-PP). Reducing PKS subclade II is characterized by enzymes missing the ER domain; PKs synthesized by PKSs of this subclade are predicted to either lack reduced alkyl groups or to contain alkyl groups whose reduction is completed by the product of an external ER domain-containing gene, as *A. terreus* lovC [43] and *P. citrinum* mlcG. The PKSs of this clade were also found to have either a condensation (CON) domain typical of nonribosomal peptide synthetases (NPSs) [43] or an entire NPS module consisting of a CON domain, and an acyl carrier or phosphopantetheine attachment site (ACP or PP) domain. The absence of a *DhPKS1* homologous gene in the less aggressive Italian isolates prompted us to further investigate the possible significance of this gene as a virulence determinant. Results from PHI-base search revealed that *DHPKS1* is very similar to *PKSs* from filamentous fungi known to be involved in the production of important pathogenicity factors. Among these, it showed 35% identity with *BcPKS6* gene of *B. cinerea* which encodes a key enzyme for botcinic acid biosynthesis, a phytotoxin involved in virulence of the fungus on tomato [44]. Moreover, *BcPKS6* belongs to a PKS cluster co-regulated by the Gα subunit BCG1, which, in turn, is essential for pathogenicity of *B. cinerea* on bean leaves [45] and is part of a clade including several PKSs responsible for the synthesis of cyclic polyketides like: LovB (LNKS, nonaketide part of lovastatin [46]); MlcA (nonaketide part of citrinin; [47]), EQS (equisetin; [48]) and FusS (fusarin; [49]). DHPKS1 has also a relatively high homology (Table 1) with the *Magnaporthe grisea* gene *ACE1* encoding a putative PKS expressed exclusively during penetration of *M. grisea* into leaves and involved in the recognition of the fungus by resistant rice varieties carrying the resistance gene *Pi33*, thus revealing a role for avirulence [50, 51]. Orthologous to *DhPKS1* is also *AbPKS9* (*DEP5*) gene involved in biosynthesis of depudecin, an 11 linear polyketide inhibitor of histone deacetylase (HDAC) virulence factor of the fungus *A. brassicicola* [52].

Among the functionally characterized PKSs *DhPKS1* orthologs, there is also *lovB* of *A. terreus*, which synthesizes the nonaketide chain of lovastatin, a 3-hydroxy-3-methylglutaryl coenzyme A (HMG-CoA) reductase inhibitor [53]. *A. terreus* has been known to carry an unusual *PKS* gene cluster for the polyketides in which two *PKS* genes (*lovB* and *lovF*) are closely linked in the cluster and are required for the biosynthesis of the nonaketide and the diketide moieties of the compound, respectively [54]. Interestingly, we also found that LovF of *A. terreus* was homologous to DhPKS24, suggesting the presence in *D. helianthi* 7/96 of a similar *PKS* gene cluster. The same hypothesis has been formulated for the biosynthesis of both zearalenone in *Gibberella zeae* [55] and the linear polyketide T-toxin in race T of *C. heterostrophus*. The synthesis of T-toxin, essential for fungal virulence on male sterile cytoplasm corn [3], seems to involve a *PKS1* together with *PKS2* [1].

In our work, as a case study, knock-out mutants of *DhPKS1* were produced by targeted DNA integration through *Agrobacterium*-mediated transformation. This method has been successfully applied to study gene functions in other phytopathogenic and toxigenic fungi [2, 56]. The presence of a single copy of the T-DNA made the analysis of the transformants straightforward. Unfortunately, it was not possible to confirm the above results by performing experiments with complementation mutants. The reason why no double mutants were obtained is unknown, but it seems that this fungal strain cannot be doubly transformed. As a result of *DhPKS1* gene inactivation, reduced fungal virulence on a susceptible sunflower cultivar was observed. Indeed, in contrast with the wild type, mutants caused less visible symptoms after artificial inoculation of fungal mycelia on stem and leaves. This indicates that *DhPKS1* could be involved in virulence of the sunflower stem canker agent. The *DhPKS1* gene disruption also seems to affect in vitro production of toxic secondary metabolites by *D. helianthi*. Liquid filtrates οf Δ*DhPKS1* mutants showed a reduced phytotoxicity on sunflower seedlings. The highly reducing synthase *DhPKS1* probably produces a metabolite, not yet identified, which could be involved in the *D. helianthi* 7/96 strain virulence on sunflower. However, for a better understanding of the possible role of *DhPKS1*, more studies should be carried out considering also the role of the other PKSs genes that cluster with it.

In previous work [26] the polyketidic metabolite phomozin, an ester of orsellinic acid, was isolated both from culture filtrates of *D. helianthi* and from infected plants, and its possible role in development of symptoms was suggested. Orsellinic acid synthase is the simplest tetraketide synthase and is grouped with the nonreducing PKSs (NR-PKSs). Our results indicate that, in the case of *D. helianthi* 7/96 orsellinc acid synthase could be coded by *DhPKS7* due to its high homology with the *A. nidulans* gene EAA59563 coding for orsellinc acid synthase in *A. nidulans* [57]. The toxinic theory is supported by the evidence that other phytopathogenic species related to the genus *Phomopsis* produce toxic metabolites involved in pathogenesis [58–60]. For a disease caused by a *Phomopsis* species affecting soybean, similar to stem canker of sunflower, the possible involvement of a phytotoxin was also implied [61]. Polyketide synthases have been reported as important virulence factors in other several phytopathogenic fungi such as *C. heterostrophus* [3], *Colletotrichum lageniarum* [62] and *A. alternata* [63]. In *C. nicotianae*, the genes *CTB1* and *CTB3* encode two polyketide synthases, involved in the biosynthesis of cercosporin, a photoactivated perylenequinone toxin, which play a key role in fungal pathogenesis [64, 65].

## Conclusions

The PKS sequences reported here are a new important resource that will be useful for further research in the biology, ecology and evolution of *D. helianthi* and in general of fungal plant pathogens. Further investigation is necessary to fully understand the role of *D. helianthi* PKS genes. Moreover, it will be very important to verify if the PKSs identified in *D. helianthi* sensu stricto are also present in other species of *Diaporthe*, which have been associated with sunflower stem canker.

## Additional file

Additional file 1: List of the 40 putative PKS in *Diaporthe helianthi* isolate 7/96 and related information. Domain analyses revealed that most *PKS* genes coded by *D. helianthi* are highly reducing PKSs, whereas only eight PKSs lack reducing domains and cluster with non-reducing PKSs. In grey are highlighted the genes partially sequenced. (XLSX 76 kb)

## Abbreviations

ACP: Acyl carrier protein
AT: Acyltransferase
DH: Dehydratase
EQS: Equisetin
ER: Enoylreductase
HDAC: Histone deacetylase
HMG-CoA: 3-hydroxy-3-methylglutaryl coenzyme A
Hph: Hygromycin phosphotransferase
KR: Ketoreductase
KS: Ketosynthase
LNKS: Nonaketide part of lovastatin
Lov: Lovastatin
MeT: Methyltransferase
NRPS: Nonribosomal peptide synthetase
PKSs: Polyketide synthases
PP: Phosphopantetheine
TE: Thioesterase
